# From responsibility to value: ESG and long-term corporate value

**DOI:** 10.1371/journal.pone.0322018

**Published:** 2025-04-23

**Authors:** Lanlin Fang, Xuemeng Guo

**Affiliations:** School of Economics and Management, Beijing Jiaotong University, Beijing, China; University of Bologna, ITALY

## Abstract

Although a large body of literature has explored the relationship between ESG and firm value, the findings are inconsistent and most studies have focused on mature Western markets, with relatively little research on ESG practices and value creation effects in emerging markets, especially in transition economies such as China. This paper analyzes the correlation between ESG performance and firms’ long-term value using data from 4,185 listed companies in China’s A-share market from 2017–2022. We find that good ESG practices can significantly enhance firms’ future cash flows, long-term value and market competitiveness. The results suggest that ESG increases long-term firm value by reducing weighted average cost and increasing return on invested capital. The effects of firms’ HHI index, nature of property rights and ISO 9001 system certification on the relationship are further investigated and it is found that Lerner’s index acts as a mediating variable to enable ESG performance to affect firms’ long-term value by enhancing their market pricing power and competitiveness. This paper serves as an important reference for corporate managers to integrate ESG concepts into strategic planning for sustainable development and long-term value growth, helping enterprises implement ESG strategies for long-term corporate success and sustainable development.

## Introductory

Against the backdrop of globalization and sustainable development, ESG (Environment, Social, Governance) has become a key indicator of long-term corporate value. International organizations and governments have introduced policies to encourage companies to implement ESG practices in order to promote the coordinated development of economy, society and environment. The Chinese government’s “dual-carbon” goal and the concept of common wealth have incorporated ESG concepts into national strategies, reflecting expectations and requirements for companies in terms of environmental protection, social responsibility and good governance. These macro policies provide a clear development direction for enterprises, as well as policy support for long-term value creation.

At the micro level, the real-life challenges and opportunities faced by enterprises have also driven the integration of ESG concepts in their business activities. As consumers and investors pay increasing attention to CSR, the ESG performance of enterprises has become an important factor affecting their brand reputation, market competitiveness, and financing costs [1–3]. By improving ESG performance, firms are able to attract more investor attention, increase employee satisfaction, and reduce operational risks, thus gaining an advantage in the fierce market competition [4,5]. However, enterprises also face problems in ESG practice such as insufficient disclosure of information and difficulty in measuring the effects of practice, the existence of which restricts the full realization of ESG value.

Although there is a large body of literature exploring the relationship between ESG and firm value, the findings are inconsistent and most of the studies limit their focus on mature Western markets, with few studies on ESG practices and value creation effects in emerging markets, especially in transition economies such as China. Existing literature focuses on the relationship between ESG disclosure quality [6,7], ESG practices and corporate risk management [8,9], but the issue of the relationship between ESG and long-term corporate value is not yet adequately investigated, and these gaps in the research offer room for further exploring the relationship between ESG and long-term corporate value.

Based on this, this study presents the hypothesis that ESG performance can affect the long-term value of firms by selecting data from China’s A-share listed companies from 2007 to 2022. This study aims to explore the intrinsic link between firms’ ESG performance and long-term value, with a special focus on the long-term value creation of firms through the impact of ESG performance on firms’ return on invested capital (ROIC), weighted average cost of capital (WACC), and firms’ future cash flows. The study hypothesizes that ESG practices can enhance ROIC by improving firms’ operational efficiency and risk management capabilities, and, at the same time, reduce firms’ cost of capital by enhancing investor and consumer trust, thus affecting the WACC. In addition, this study also examines the direct impact of ESG performance on firms’ future cash flows, and the role of the Lerner Index as a mediating variable in the relationship between ESG performance and firms’ long-term value relationship. ESG performance is found to significantly and positively affect ROIC and firms’ future cash flows, but negatively affect WACC, with the Lerner index playing a mediating role in this process.

This study delves into the intrinsic link between firms’ ESG performance and long-term value, in particular how ESG performance shapes firms’ long-term value by affecting the difference between firms’ return on invested capital (ROIC) and weighted average cost of capital (WACC). This paper focuses on the following questions: first, how is ESG performance related to long-term firm value (ROIC-WACC)? Second, how does ESG performance affect firms’ long-term cash flows? Finally, does the Lerner index, as a mediating variable, play a significant mediating role in the relationship between ESG performance and firms’ long-term value? The important contribution of this study is to reveal the complex relationship between ESG performance and firms’ long-term value and to clarify the impact of ESG performance on firms’ ROIC, WACC, and firms’ future cash flows. The findings suggest that ESG practices not only enhance firms’ profitability and cash flows, but also reduce the cost of capital, which together contribute to the enhancement of firms’ long-term value. In addition, using the Lerner index as a mediating variable reveals a new perspective on how ESG performance indirectly affects long-term corporate value by influencing market structure and corporate pricing power. Unlike previous studies, this paper provides an in-depth analysis to better understand how ESG creates value for firms in the long run under different environments and market conditions, and provides strategic recommendations for firms to achieve long-term sustainable development.

## Literature review

### ESG and corporate economic consequences

Academic research on the economic consequences of ESG performance of Chinese firms is in the initial exploration stage. Previous studies have shown that ESG has a significant effect on corporate performance [10,11] and increases stock returns [12,13]. However, in view of the fragmented conclusions of related studies, scholars’ debate is far from settled regarding the sustainable impact of ESG on enterprises and its economic consequences economic consequences.

Most of the existing literature on the relationship between ESG management and long-term corporate value centers around the relationship between ESG and corporate performance. Qiu and Yin (2019) found that the explanatory power of ESG indicators surpasses that of traditional financial indicators in assessing long-term corporate value, with the former providing a more comprehensive picture of a firm’s overall performance and potential risks [2]; Ghoul et al. (2017) also pointed out that ESG indicators have high explanatory power in predicting long-term corporate performance [14]. Li Jinglin et al. (2021) showed that the explanatory power of ESG indicators for the long-term value of Chinese listed companies is significantly better than that of traditional indicators [15]. Some scholars have also studied the correlation between different dimensions of ESG and the long-term value of enterprises through the method of empirical research [16] The results show that compared to a single ESG indicator, the positive correlation between the comprehensive ESG scores and the long-term value of the enterprise is more significant. So, it is more effective to use the composite ESG score as an indicator for long-term corporate value assessment. These studies have provided strong evidence for the application of ESG management in enterprise value assessment, and also pointed out that future research needs to further explore the complexity and diversity of the mechanisms of ESG risk and long-term enterprise value.

When exploring the relationship between ESG performance and long-term corporate value, existing research often fails to fully reveal the intrinsic link between the two, mainly due to insufficient consideration of two key elements: the dynamics of corporate value growth and the multidimensionality of ESG performance. Enterprise value growth is a dynamic process involving multiple factors and stages, which is not only affected by the current financial performance of the enterprise, but also closely related to the enterprise’s future development potential, market competitiveness, and innovation ability. These factors closely affect a company’s ESG performance, and the multidimensional nature of ESG performance, including environmental protection, social responsibility, and corporate governance, exerts a multifaceted impact on corporate value. Moreover, the costs and benefits of an enterprise’s ESG investments exhibit nonlinear characteristics at different stages, which requires enterprises to weigh their long-term benefits against short-term financial pressures when formulating their ESG strategies. In the short term, firms may need to invest in upgrading environmental standards or strengthening social responsibility programs, which may put pressure on their financial position. However, in the long term, these investments can enhance a company’s brand image, strengthen consumer loyalty, and attract and retain talent, resulting in a more sustainable competitive advantage and value growth. Therefore, supplementing the research on the relationship between ESG and long-term corporate value helps to reveal the impact of ESG on the long-term benefits of corporations, and provides theoretical support for corporations to implement sustainable development strategies.

### ESG and long-term corporate value

Enterprise value is usually defined as the present value of a firm’s future profitability, which not only includes the profitability of existing assets, but also reflects the firm’s strategic adaptability to the operating environment [17,18]. Most studies have confirmed the correlation between ESG performance and enterprise value, including positive correlation, negative correlation, or “U” shape relationship [19,20]. Among all factors studied, CSR and people-centered values are considered to have a particularly positive impact on both financial and non-financial corporate value. In turn, a firm’s value not only affects its internal management and employee behavior, but is also key to building trust and relationships with external stakeholders.

Due to the difference in the starting point of research, there are differences in the definition of enterprise value by foreign scholars. Since the macro environment and governance environment faced by domestic enterprises are quite different from that of foreign countries, scholars have different views on the composition of enterprise value. Lu Zhengfei and Shi Yu (2002), from the perspective of market exchange, believe that enterprise value is the comprehensive performance of the enterprise’s adaptation to the market environment, profitability and duration of competitive advantage, which not only measures the profitability of the enterprise’s existing assets, but also embodies the enterprise’s strategic adaptability to the operating environment [21]. This definition is actually a process of recognizing and evaluating the sustainable development potential of an enterprise with the integration of various factors such as the enterprise’s ability, macroeconomic situation and subjective human needs. Most studies consider that enterprise value is not the book value of the existing assets of an enterprise, but the market exchange value based on the intrinsic value of the enterprise. Therefore, enterprise value appraisal studies the fair market value of an enterprise’s assets as a whole with not only consideration of the current market conditions, but also in view of the enterprise’s overall future profitability.

To summarize, ESG factors, as key variables affecting the long-term value of enterprises, have received extensive attention from both academia and practice in terms of the mechanism and path of their influence on enterprise value. While pursuing economic benefits, enterprises should also focus on their construction in terms of social responsibility and governance structure in order to realize sustainable development and long-term value growth.

## Theoretical explanations and research hypotheses

### ESG and the “sustainability” of corporate value

Though the risk of damage to enterprise value has increased, long-term investors GPIF focus on ESG remain intensive. In order to understand this ascending trend, it is important to clarify the relationship between ESG and enterprise value. The environment surrounding business has changed considerably in recent years. Events such as technological advances, increased regulation, and unexpected crises (disasters, financial and political changes) have made business decisions more difficult than ever. From a long-term value perspective, if ESG determines the long-term value of a company, investors need to estimate long-term value by measuring ESG outcomes. If firms disclose measures of long-term value, the market will focus on those measures rather than on short-term earnings. Early research on firm value depended on more than just financial factors, and Kaplan and Norton (1992) proposed that if ESG is used as a driver of a firm’s future financial performance, “balanced scorecard” can be complemented by operational measures such as customer satisfaction, internal processes, and the organization’s innovation and improvement activities [22]. According to previous research, all the factors of complementary financial measure, revenue growth, return on invested capital and effective investor communications can drive/facilitate market capitalization. However, the biggest challenge facing Chinese companies today is optimizing returns and achieving high-quality growth. For listed companies seeking to attract long-term strategic investors, the core of market capitalization management is to cut through the fog of the capital market and return to the fundamentals of corporate value creation, i.e., to use Return on Invested Capital (ROIC) as a measure of long-term corporate value to examine corporate development from an internal perspective [23].

In past research, the focus has predominantly been on the relationship between the impact of ESG and the economic consequences for enterprise value. However, essential investigations into the sustainable development perspective of ESG have been lacking. By analyzing the influence of ESG on firms from a long-term development vantage point, it becomes possible to quantitatively measure firms’ current and target values, thereby revealing the enhancement of firm value by ESG and its sustainable impact on long-term value.

The ESG value effect has been widely deliberated in existing literature, with the majority of studies endorsing the notion that ESG performance positively influences firm value. Friede et al. (2015), after scrutinizing over 2,200 studies since 1970, ascertained that according to over 90% of those studies, firms’ ESG performance can engender a positive and sustainable value effect on their long-term development [24]. Nevertheless, academic viewpoints diverge regarding the specific mechanism underlying the ESG value effect. Some studies accentuate the contribution of the externality aspect of ESG performance to firm value, while others concentrate on the role of ESG behaviors in internal value creation within the firm.

Beyond ESG’s contributions to enhancing firm external value and facilitating internal value creation, the ESG value effect is also perceived as a process of procuring external resources. It is posited that enterprises’ investment in ESG aids in augmenting their market credit and reputation resources, enabling them to garner support and resources from stakeholders [25,26]. The ESG index serves as a significant risk contributor to cross-country risk spillovers, signifying its high sensitivity to climate transition. This further implies that investors and policymakers must closely monitor climate change and climate transition risks and enhance the transparency and efficiency of ESG, which is pivotal for mitigating climate risk and fostering sustainable corporate development [27]. Additionally, certain studies zero in on the role of ESG behaviors in internal corporate value creation. Corporate ESG performance chiefly promotes value enhancement through mechanisms such as augmenting corporate innovation investment and attracting analyst follow-up. Moreover, internal innovation investment and external analyst attention also exert a synergistic effect in bolstering corporate value [17].

Nonetheless, some scholars have reservations about the ESG value effect. They contend that ESG investments might sacrifice a firm’s current strategic resources, potentially harming its market performance [28]. Halkos et al. (2016) even pointed out that substantial ESG investments could crowd out a firm’s limited cash flow and amplify operational risks [29].

Recently, empirical literature has proposed that there may be a nonlinear relationship between sustainability practices and firm value [30,31]. In the initial stage, as enterprises increase their investments in ESG, they may need to allocate substantial resources to improve the environment, fulfill social responsibilities, and optimize governance structures. This could lead to a rise in short-term costs and have a certain negative impact on firm value. For example, to meet higher environmental standards, an enterprise might have to purchase expensive environmental protection equipment or conduct large-scale renovations of its production processes, which would increase capital expenditures and thus reduce the enterprise’s profits and value in the short term.

However, once the ESG investment exceeds a certain threshold, the enterprise will begin to reap the long-term benefits brought about by sustainability practices. From the environmental dimension, an enterprise’s environmental protection measures may result in improved resource utilization efficiency. For instance, energy costs can be reduced through energy conservation and emission reduction, or new market demands can be tapped into by developing environmentally friendly products, thereby increasing the enterprise’s revenues and profits [32]. In the social dimension, actively fulfilling social responsibilities helps enhance the enterprise’s brand image and employee satisfaction, which in turn improves customer loyalty and employee productivity, creating more value for the enterprise. From the governance perspective, a sound corporate governance structure can reduce agency costs, improve decision-making efficiency, and enhance the enterprise’s ability to cope with risks, facilitating stable development and value enhancement in the long run [33].

Nevertheless, current research has not fully considered this nonlinear relationship in the construction of theoretical frameworks. Most studies, when exploring the link between ESG and firm value, tend to assume a simple linear association, failing to comprehensively capture the complex dynamic process of changes in firm value at different ESG investment levels. This limitation leads to certain biases in existing research when explaining and predicting the value creation process of enterprises through ESG practices, preventing them from providing precise decision-making guidance for enterprises. Therefore, future research needs to further refine the theoretical framework by incorporating the nonlinear relationship between sustainability practices and firm value, in order to gain a deeper understanding of the impact mechanism of ESG on the long-term value of enterprises and offer more targeted and effective suggestions for enterprises when formulating ESG strategies. By constructing a theoretical model that encompasses ESG investment levels, cost-benefit changes, and dynamic changes in firm value, and by analyzing the key factors and interactions at different stages, a more accurate explanation and prediction of the impact of ESG practices on firm value can be achieved. Additionally, further research can explore how to determine the optimal level of ESG investment and how to better balance short-term costs and long-term benefits under different industries and enterprise scales to maximize firm value.

### Application of the ESG-ROIC model

The process of achieving sustained growth and medium to long term enterprise value enhancement needs to begin with the definition of long term enterprise value enhancement: ‘ROIC> WACC’ [34]. Through the decomposition of the ROIC factorization above, the cost of capital component of this can be further categorized. Assuming that the firm is going concern, the cost of capital should be “WACC-perpetual growth rate”. As one component of WACC, the shareholder cost of capital is expected to be reduced by reducing the risk of the firm, either through beta stabilization or by adjusting the individual risk premium. In financial theory, the higher the perpetual growth rate is, the lower the cost of capital and the higher the value of the firm.

Therefore, “enterprise value enhancement” can be defined as “ROIC (NOPAT/ invested capital)> cost of capital (WACC - perpetual growth rate)”. Enterprises can effectively reduce their WACC by strengthening internal financing constraints and improving corporate governance mechanisms. From the perspective of the entire source of funds of an enterprise, this end is impossible to be achieved by using a single financing method, but by a combination of various financing methods. Therefore, the total cost of capital of an enterprise cannot be determined by a single cost of capital, but requires the calculation of a comprehensive cost of capital. The weighted average cost of capital (WACC) serves as an important reference standard for corporate decision-making, especially in investment decisions. Enterprises can compare the expected rates of return of an investment project with WACC to determine whether the investment project can create sufficient value [35,36]. By adjusting the ratio of equity and debt, the WACC is lowered, thus reducing the cost of financing and improving the profitability of the enterprise. If the return of a project is higher than the WACC, the project has a positive NPV and has a positive impact on firm value. Resultantly, investors may be more willing to pay a higher price or accept a lower rate of return for firms with higher ESG scores because their investments seem less risky and more sustainable.

This leads to hypothesis H1

H1: A good ESG score implies increased long-term corporate value.

### ESG and corporate future cash flows

Risk factors across ESG topics may undermine the ability to generate free cash flow over the medium to long term. When capturing these risk factors, institutional investors need not only assess the risks of business owned by firms from an ESG perspective, but also reorganize their own risks from an ESG perspective and align their perspectives with those of other institutional investors who have been operating for a long period of time. However, previous research is lacking in examining how ESG performance affects firms’ investment cash flows and WACC from an internal perspective. In order to maintain the stability of ESG performance, firms may need to sustain long-term responsible investments, such as environmental technology up-gradations, employee training and welfare improvements [37,38]. These investments tend to have long payback periods and uncertainty, and require firms to have sufficient cash reserves to support them. Therefore, ESG-oriented firms may tend to hold higher levels of cash to cope with the demands and uncertainty risks of responsible investments.

The corporate investment decision is critical to the sustainability and growth of a company. This decision involves allocating funds to various projects and investments in order to maximize shareholder value. Indeed, this decision is often challenging and is further complicated by constraints in accessing external funds due to information asymmetry or agency problems that lead to the emergence of personal interests within the firm. Among other things, the corporate decision also affects the cash flow in investing activities, which is determined by whether inflows can increase the firm’s capital and improve its solvency. Cash flow in investment is also used as one of the important indicators for investors to assess the value and investment potential of a company and has a significant impact on the market evaluation of the company.

Positive ESG performance can reduce inefficient investment by effectively mitigating agency problems and reducing short-sightedness on the part of managers. By proactively disclosing information about the enterprise’s environmental, social, and governance areas, the enterprise can communicate its commitment to sustainable development and social responsibility to society, thus, an implicit contract and multi-stakeholder constraints on managers are formed, so as to avoid self-interested decision-making by managers [39], which will motivate the enterprise to focus on the long-term interests, reduce shortsightedness, and avoid inefficient investment. On the other hand, ESG disclosure can play the role of reputation surveillance mechanism, forcing enterprises to pay due attention to long-term interests and avoid short-sighted behavior. If the enterprise is exposed to short-sighted behavior, the enterprise’s supply chain relationship will be negatively affected, and this risk will force managers to reduce opportunistic tendencies in daily operations and shift to focusing on the realization of the long-term value of the enterprise, which will promote the enterprise’s investment activities and improve the enterprise’s future cash flow [40].

Corporate ESG performance mainly affects the level of corporate cash flow through two ways: one is to directly change the key items of the income statement and thus directly affect corporate cash flow; the other is a non-direct way, i.e., through the influence of some processes or elements in the operation of the enterprise, the financial data of the enterprise can be affected, thus indirectly changing the cash flow of the enterprise. Long-term focus on ESG performance of the enterprise can reduce corporate costs, and the ESG factors of environmental, social and corporate governance dimensions have different degrees of influence on the different subject enterprises [41], which manifests in all aspects of the enterprise, including enterprise’s earnings before interest and taxes, taxes, depreciation and amortization, changes in working capital and capital expenditures, etc., thus affecting the level of future cash flow [42].

This leads to Hypothesis 2 in this paper

H2: ESG performance is positively associated with firms’ future operating cash flow forecasts.

## Research design

### Sample selection

Based on H1, this study selects 4,185 companies listed on China’s A-share market from 2007 to 2022 as the research sample, and adopts data such as CNRDS ESG ratings and CSMAR listed companies. Meanwhile, listed companies in the financial industry, listed companies in ST and *ST, samples of companies with missing main data or key variables, listed companies with missing financial data are excluded. Considering the possible impact of outliers, all continuous variables are winsorized at 1% and 99% levels, and the final sample data is 32,826 observations.

### Variable selection

#### Dependent variable.

Long-Term Enterprise ValueLong-Value_i,t_ = ROIC_i,t_ -WACC_i,t_. where ROIC_i,t_ denotes the enterprise value creation capacity of firm i in year t, measured by using (share price per share × number of shares)/total assets, and WACC_i,t_ denotes the weighted average cost of firm i in year t, measured by using (market value of the firm’s equity/percentage of equity to total financing) × cost of debt cost + (market value of firm’s debt/debt as a percentage of total financing) x cost of debt x (1-corporate tax rate).

The firm’s future cash flow FCF_i,t_. represents the future operating cash flow of firm i in year t. It is measured by using data on the cash flows incurred by the firm from operating activities such as fixed assets or financial instruments.

#### Independent variable.

Referring to the existing literature [43], ESG ratings denote the ESG ratings of firm i in year t. This paper adopts CNRDS database collection. Compared with other domestic ESG evaluation systems, CNRDS ESG database design is more scientific. In the construction of reference to the MSCI ESG Stats Database, combined with the actual situation of Chinese enterprises to measure the ESG situation of listed companies, CNRDS ESG database can intuitively express the characteristics of different dimensions of corporate social responsibility.

#### Control variable.

Considering that some factors may have an impact on the results of corporate ESG ratings as well as future cash flows, this paper refers to the existing research [20,21,44], and sets the control variables of the model in terms of the basic characteristics of the firms as well as the performance of the capital market. The control variables include gearing ratio, operating income growth rate, management shareholding, fixed assets ratio, number of directors, proportion of independent directors, current liability ratio, year of listing of the firm, proportion of shares held by the first largest shareholder, and the control of the respective fixed effects of year and industry. ε_i,t_ denotes the random error term. In addition, this paper further controls for year fixed effects and industry fixed effects to control for differences in firms’ characteristics due to time changes or industry differences. The specific variable definitions as well as the measurements are shown in Table 1.

**Table 1:** Definitions and descriptions of key variables.

Variable type	Variable name	Notation	Calculation method
Explanatory variable	Long-term enterprise value	Long-Value	ROIC-WACC
	Future cash flows of the enterprise	FCF	Operating cash flow of the enterprise under the indirect method
Explanatory variable	Corporate ESG Ratings	ESG	CNRDS ESG rating
Control variable	Enterprise size	Size	Natural logarithm of total assets of the company at the end of the period
	gearing	Lev	Ratio of total liabilities to total assets of the enterprise
	Current liabilities ratio	Cl	Ratio of current liabilities to total assets of the enterprise
	Year of listing	Lnlistage	Logarithmic number of years of listing
	Revenue growth rate	Growth	Sales revenue growth rate
	Management shareholding	Managehold	Management shareholding
	Number of Directors	Board	Number of Board of Directors
	Proportion of independent directors	IndDir	Proportion of independent directors to all directors
	Shareholding ratio of the largest shareholder	Top1	Ratio of the number of shares held by the largest shareholder of the enterprise to the total number of shares held
	Fixed assets ratio	PPE	Net fixed assets/ total assets

### Empirical model

In order to test the impact of ESG performance on long-term corporate value in Hypothesis 1, with reference to existing related studies [45], this paper establishes the following multiple linear benchmark regression model:


Long−Valuei,t = α0 + α1ESGi,t+αControlsi,t+i.Year+i.Ind+εi,t (1)

In order to test the impact of ESG on the future operating cash flow of enterprises in Hypothesis 2, this paper establishes the following multivariate linear benchmark regression model:


FCFi,t = β0 + β1ESGi,t+βControlsi,t+i.Year+i.Ind+εi,t (2)

The choice of using one-period lagged corporate operating cash flows as an explanatory variable is to address possible endogeneity issues, particularly reverse causality and simultaneity bias. Based on economic logic and data characteristics, taking into account delays in information acquisition and decision-making, as well as considering management’s operational decisions based on historical cash flow information, lagged variables provide a more accurate picture of the time-series dynamics of the impact of firms’ cash flows on their operating activities.

## Empirical analysis

### Descriptive statistics

As shown in the descriptive statistics in Table 2, the volatility of firms’ years of listing and business growth rates further emphasizes the sample firms’ operating conditions in different stages of development and competitive market environments. These statistical analysis provides rich background information for an in-depth analysis of ESG’s impact on business performance, financial health, and operational efficiency of firms, and thereby provides a valuable foundation for subsequent research.

**Table 2:** Descriptive statistics of the main variables.

Variables	Sample	Mean	Med	Std	Min	Max
Long-Value	32826	−5.769	−5.667	1.225	−8.984	−3.100
FCF	32826	0.0520	0.0500	0.0660	−0.150	0.244
ESG Score	32826	26.20	23.67	11.01	7.405	58.75
Board	32826	8.546	9	1.684	5	15
IndeDir	32826	0.374	0.333	0.0530	0.300	0.571
Manahold	32826	15.92	2.460	20.79	0	69.50
Lev	32826	0.394	0.386	0.196	0.0490	0.860
Size	32826	22.11	21.89	1.273	19.89	26.23
Top1	32826	34.40	32.35	14.76	8.700	74.57
Growth	32826	0.310	0.121	0.773	−0.679	5.554
Lnlistage	32826	1.849	1.946	0.943	0	3.332
Cl	32826	0.826	0.882	0.172	0.264	1
PPE	32826	0.207	0.176	0.153	0.0025	0.685

### Empirical testing

#### Benchmark regression results.

The long-term value of a firm is closely related to its economic profit, which is usually determined by the difference between the return on investment (ROIC) and the weighted average cost of capital (WACC). In order to examine the impact of ESG on firms’ long-term value and firms’ future cash flows, this paper conducts regressions based on econometric models (1) and (2) for the full sample. Table 3 reports the regression results of ESG on firms’ long-term value. Among them, the explanatory variable is long-term corporate value, and the regression results in each column are corrected for standard errors by using industry-level clustering effects; Model11 and Model13 do not include control variables, and their empirical results are 0.0229 and 0.0005, respectively, which are significant at the 1% level. After control variables being added, the results of Model12 and Model14 are 0.0059 and 0.0003, respectively, also significant at 1% level. This indicates that ESG performance can effectively improve the long-term corporate value and future cash flow. Resultantly, the regression results verify Hypothesis 1 and Hypothesis 2. In the regression coefficients of control variables in Table 3, some of the control variables also demonstrate a significant effect on the long-term value of the enterprise and the future cash flow of the enterprise. The results show that the proportion of shares held by the first largest shareholder and the size of the company have a significant positive effect on the long-term value of the enterprise and the future cash flow. This result implies that the larger the firm size is, the stronger the ability to realize long-term value enhancement and the more adequate the cash flow. This suggests that by enhancing ESG practices, firms are able to utilize their investments more effectively and improve their profitability and long-term value growth. It has proven that companies can improve their financial metrics through enhanced ESG performance, which can positively impact their long-term value and future cash flows.

**Table 3:** Benchmark regression results.

	Model11	Model12	Model13	Model14
	Long-Value	Long-Value	FCF	FCF
ESG_Score	0.0229^***^	0.0059^***^	0.0005^***^	0.0003^***^
	(0.0006)	(0.0007)	(0.0000)	(0.0001)
Board		0.0121^**^		−0.0001
		(0.0057)		(0.0004)
IndeDir		−0.0781		−0.0087
		(0.1573)		(0.0126)
Manahold		−0.0005		0.0001^**^
		(0.0004)		(0.0000)
Lev		1.1300^***^		−0.0604^***^
		(0.0517)		(0.0039)
Size		0.1523^***^		0.0072^***^
		(0.0093)		(0.0007)
Top1		0.0035^***^		0.0003^***^
		(0.0006)		(0.0000)
Growth		0.0150^**^		−0.0029^***^
		(0.0072)		(0.0007)
Lnlistage		−0.0616^***^		0.0007
		(0.0091)		(0.0008)
Cl		0.1109^**^		0.0167^***^
		(0.0452)		(0.0035)
PPE		0.2113^***^		0.0942^***^
		(0.0616)		(0.0047)
_cons	1.3630^***^	−2.2184^***^	0.0374^***^	−0.1315^***^
	(0.0171)	(0.2041)	(0.0009)	(0.0159)
N	32826	32826	32826	32826
r2_a	0.0423	0.5145	0.0082	0.1130

Note:

* ,

** , and

*** indicate significant at the 10%, 5%, and 1% levels, respectively, and the values in parentheses are cluster robust standard errors, below.

### Robustness check

#### Substitution of dependent variables.

According to Hypothesis 1, in financial analysis, Long-Value is usually understood as the present value of the firm’s expected future cash flows, while ROIC (Return on Investment) and WACC (Weighted Average Cost of Capital) are key financial indicators for assessing a firm’s ability to create value. The formula “Long-Value = ROIC - WACC” is used here as a simplified model to assess a firm’s ability to create value relative to its cost of capital. Ideally, if a firm’s ROIC is higher than its WACC, it means that the firm is generating returns in excess of its cost of capital, thereby creating value for shareholders. Conversely, if ROIC is below WACC, the firm is eroding shareholder value.

ROIC: Based on previous research [46], this paper takes the return on investment (ROIC) of a firm as a key indicator for measuring the long-term value of a firm. According to the results of Model21 in Table 4, we found that there is a positive association between a firm’s ESG performance and ROIC, and the coefficient of this relationship is 0.0003. This suggests that as a firm’s ESG performance improves, it is able to utilize its investment more efficiently in generating returns, which in turn improves the firm’s ROIC. Good ESG practices can improve operational efficiency, enhance brand value, lower the cost of capital, reduce legal and compliance risks, promote innovation and competitive advantage, increase employee satisfaction and productivity, enhance market trust and stability, and take a long-term perspective on investment [45,47,48]. Together, these factors help firms to be effective in reducing costs, increasing revenues, and improving market value, which leads to higher financial returns on invested capital and ensures sustained growth and long-term success. This result supports the hypothesis that ESG practices can enhance the long-term value of firms.

**Table 4:** Long-value robustness test.

	Model21	Model22	Model23	Model24	Model25
	ROIC	WACC	Long-Va	TobinQ	Non-liner
ESG_Score	0.0003***	−0.0055***		0.0027**	0.0069***
	(0.0001)	(0.0007)		(0.0013)	(0.0026)
Board	−0.0002	−0.0128**	0.0127**	−0.0008	0.0121**
	(0.0005)	(0.0057)	(0.0058)	(0.0111)	(0.0057)
IndeDir	−0.0419***	0.0218	−0.0788	0.8283***	−0.0777
	(0.0141)	(0.1567)	(0.1587)	(0.3023)	(0.1574)
Manahold	0.0001***	0.0007	−0.0004	−0.0043***	−0.0005
	(0.0000)	(0.0004)	(0.0004)	(0.0008)	(0.0004)
Lev	−0.0924***	−1.2398***	1.1217***	−1.1595***	1.1295***
	(0.0046)	(0.0510)	(0.0522)	(0.1272)	(0.0518)
Size	0.0159***	−0.1336***	0.1553***	−0.2819***	0.1522***
	(0.0008)	(0.0093)	(0.0093)	(0.0249)	(0.0092)
Top1	0.0004***	−0.0031***	0.0036***	−0.0018*	0.0035***
	(0.0000)	(0.0005)	(0.0006)	(0.0011)	(0.0006)
Growth	0.0015**	−0.0121*	0.0140*	0.0070	0.0150**
	(0.0007)	(0.0070)	(0.0072)	(0.0134)	(0.0072)
Lnlistage	−0.0095***	0.0514***	−0.0535***	0.2812***	−0.0617***
	(0.0008)	(0.0090)	(0.0092)	(0.0197)	(0.0091)
CL	0.0342***	−0.0770*	0.1080**	−0.3388***	0.1107**
	(0.0037)	(0.0452)	(0.0456)	(0.0989)	(0.0452)
PPE	−0.0164***	−0.2257***	0.2478***	−1.0738***	0.2118***
	(0.0050)	(0.0613)	(0.0617)	(0.1175)	(0.0616)
ESGmean			0.0044***		
			(0.0012)		
c.ESG_Score# c.ESG_Score					−0.0000
					(0.0000)
_cons	−0.2676***	9.6321***	−2.3483***	8.6055***	−2.2280***
	(0.0191)	(0.2060)	(0.2097)	(0.5075)	(0.2059)
N	32826	32826	32826	32451	32826
r2_a	0.1384	0.5199	0.5127	0.2245	0.5145

Note: *, **, and *** indicate significant at the 10%, 5%, and 1% levels, respectively, and the values in parentheses are cluster robust standard errors.

WACC: The results in Table 4 show that there is a negative relationship between ESG performance and WACC with a coefficient of -0.0055, implying that those firms with higher ESG scores are able to obtain funding at a lower cost. This may be due to the fact that market participants have higher confidence in the long-term stability and risk management ability of these firms and are therefore more willing to provide funds at lower rates. WACC, as a measurement tool of the cost of financing a firm, reflects the average cost of obtaining funds from all sources of capital. The results show a negative correlation between ESG performance and WACC, which implies that the higher a firm’s ESG score is, the lower its cost of financing. The logical basis of this result lies in the fact that good ESG performance enhances the reputation and credit standing of firms, allowing them to obtain debt and equity financing at lower interest rates in the capital market [49]. In addition, firms with good ESG performance may be favored by investors because they are perceived as less risky investments, which further reduces the firm’s cost of capital.

Tobin Q: In the context of ESG research, using Tobin Q as a proxy for long-term value in robustness tests helps to validate the relationship between ESG performance and firm value. If the results remain consistent when Tobin Q is used instead of other long-term value measures, it provides stronger evidence that the observed impact of ESG on firm value is robust and not sensitive to the specific metric chosen. We can see in Table 4 that the Tobin Q coefficient is 0.0027 and is significant at the 5% level. To summarize, enhanced ESG performance correlates with a lower weighted average cost of capital (WACC). This reflects the market’s augmented trust, leading to cheaper capital, thus cutting firms’ financing costs. When return on invested capital (ROIC) surpasses WACC (i.e., ROIC - WACC > 0), firms earn economic profits beyond covering costs, which can be reinvested for growth. This bolsters their market competitiveness and industry standing. Over time, such value creation fuels long-term firm value growth, yielding stable shareholder returns and luring more investors, solidifying market value. Essentially, firms can use capital efficiently and access it cheaply, signaling value creation. Cumulatively, this capacity augments long-term firm value, as corroborated by the decomposition of long-term value, highlighting that sound ESG performance uplifts firm value.

Firms’ future operating cash flows under the direct method: in order to verify the robustness of the relationship between ESG performance and firms’ future, this paper shifts the focus of the study to the forecasting of firms’ future operating cash flows under the direct method. In traditional analyses, operating cash flows are often forecasted by using the indirect method, which adjusts for changes in non-cash items and working capital from net income. In the robustness test, we switch to the direct method, i.e., predicting future cash flows by analyzing the raw data of cash inflows and outflows of the firm. According to the results, the relationship between ESG performance and cash flows remains significant even when the direct method is used to predict future cash flows, with a coefficient of 0.0003 and significance at the 1% level (Model26). This suggests that ESG practices not only affect firms’ profitability and financing costs, but also directly correlate with firms’ ability to generate cash flows, which is a key driver of firms’ long-term value, thus validating the robustness of the results of Hypothesis 2.

#### Substitution of dependent variables.

In order to more accurately measure the ESG performance of firms, we take the raw ESG data scores from seven mainstream ESG rating agencies (Refinitiv, MSCI, WIND, FTSE Russell, WASDE, Shangdao Ronglv, and CNRDS) and standardizes these scores to a range from 0 to 100, and then calculates the average of these scores as a measure of firms’ ESG performance of a composite proxy variable. The purpose of this approach is to reduce the potential bias of a single rating agency’s score while capturing the different views and evaluation criteria of corporate ESG performance in the market. This approach results in a more comprehensive and balanced ESG score that more effectively represents both the current divergence and consensus among mainstream rating agencies. Averaging ESG scores from different sources helps to reduce measurement errors due to methodological differences, sample selection or subjective judgments of a single rating agency. Such composite scores can better reflect the overall market perception of corporate ESG performance and enhance the robustness of the findings. In Table 4 and Table 5, the coefficients of the mean ESG scores on long-term firm value and future firm cash flows are 0.0044 and 0.0004, respectively, both statistically significant at the 1% level. This provides more reliable evidence for analyzing the relationship between ESG performance, firm value, and future operating cash flows.

**Table 5:** Cash flow robustness test.

	Model26	Model27	Model28	Model29
	Substitution of dependent variable	Substitution of independent variable	the sustainable growth rate	Non-liner
ESG_Score	0.0003***		0.0002***	0.0002
	(0.0001)		(0.0001)	(0.0002)
Board	−0.0001	−0.0001	−0.0009*	−0.0001
	(0.0004)	(0.0004)	(0.0005)	(0.0004)
IndeDir	−0.0087	−0.0092	−0.0153	−0.0087
	(0.0126)	(0.0126)	(0.0153)	(0.0126)
Manahold	0.0001**	0.0001**	0.0001**	0.0001**
	(0.0000)	(0.0000)	(0.0000)	(0.0000)
Lev	−0.0605***	−0.0605***	0.0181**	−0.0604***
	(0.0039)	(0.0039)	(0.0071)	(0.0039)
Size	0.0072***	0.0072***	0.0109***	0.0072***
	(0.0007)	(0.0007)	(0.0012)	(0.0007)
Top1	0.0003***	0.0003***	−0.0001**	0.0003***
	(0.0000)	(0.0000)	(0.0000)	(0.0000)
Growth	−0.0029***	−0.0030***	0.0019*	−0.0029***
	(0.0007)	(0.0007)	(0.0011)	(0.0007)
Lnlistage	0.0007	0.0011	−0.0113***	0.0007
	(0.0008)	(0.0008)	(0.0008)	(0.0008)
CL	0.0166***	0.0168***	0.0184***	0.0167***
	(0.0035)	(0.0035)	(0.0048)	(0.0035)
PPE	0.0943***	0.0954***	−0.0299**	0.0942***
	(0.0047)	(0.0046)	(0.0116)	(0.0047)
ESGmean		0.0004***		
		(0.0001)		
c.ESG_Score# c.ESG_Score				0.0000
				(0.0000)
_cons	−0.1316***	−0.1416***	−0.1543***	−0.1304***
	(0.0159)	(0.0161)	(0.0233)	(0.0160)
N	32826	32826	30180	32826
r2_a	0.1129	0.1123	0.0394	0.1130

Note: *, **, and *** indicate significant at the 10%, 5%, and 1% levels, respectively, and the values in parentheses are cluster robust standard errors.

#### Endogeneity test.

In the research on the relationship between corporate ESG performance and long-term value as well as future cash flows, endogeneity issues may interfere with the accuracy of the conclusions. To address this problem, this paper adopts the instrumental variable method and selects the ESG data of other firms in the same industry (Av_ESGcnrds) as the instrumental variable. The relevant test results are presented in Table 6.

**Table 6:** Endogeneity test.

	(1)	(2)	(3)	(4)
	First-stage regression: ESG_Score	model32	First-stage regression: ESG_Score	model34
Av_ESGcnrds	0.9635***		0.9635***	
	(0.0151)		(0.0151)	
Board	0.0711	0.0088	0.0711	−0.0002
	(0.0672)	(0.0059)	(0.0672)	(0.0004)
IndeDir	1.0176	−0.1170	1.0176	−0.0099
	(1.8409)	(0.1589)	(1.8409)	(0.0125)
Manahold	0.0087*	−0.0006	0.0087*	0.0001**
	(0.0046)	(0.0004)	(0.0046)	(0.0000)
Lev	1.8248***	1.1740***	1.8248***	−0.0590***
	(0.5347)	(0.0517)	(0.5347)	(0.0039)
Size	1.1910***	0.1371***	1.1910***	0.0067***
	(0.0993)	(0.0095)	(0.0993)	(0.0007)
Top1	−0.0074	0.0034***	−0.0074	0.0003***
	(0.0063)	(0.0006)	(0.0063)	(0.0000)
Growth	0.1527*	0.0167**	0.1527*	−0.0029***
	(0.0795)	(0.0072)	(0.0795)	(0.0007)
Lnlistage	0.8244***	−0.0810***	0.8244***	0.0001
	(0.1026)	(0.0092)	(0.1026)	(0.0008)
CL	0.3557	0.1322***	0.3557	0.0174***
	(0.5131)	(0.0456)	(0.5131)	(0.0035)
PPE	2.8424***	0.0779	2.8424***	0.0900***
	(0.6964)	(0.0638)	(0.6964)	(0.0048)
ESG_Score		0.0205***		0.0007***
		(0.0014)		(0.0001)
N	32826	32826	32826	32826
r2_a		0.1102		0.0632

Note: *, **, and *** indicate significant at the 10%, 5%, and 1% levels, respectively, and the values in parentheses are cluster robust standard errors.

In the first-stage regression, the regression coefficient of Av_esgcnrds on the firm’s own ESG_Score is 0.9635, which is highly significant at the 1% significance level. This indicates a strong correlation between the ESG data of the same industry and the firm’s ESG score, providing a solid basis for its validity as an instrumental variable. Meanwhile, other control variables also participate in the regression. Each of them reflects the potential influence of firm characteristics on the ESG score from different aspects. In the second-stage regression, the regression coefficient of the firm’s ESG_Score with respect to Long-Value is 0.0205 exhibiting a highly significant level at 1%. Similarly, the regression coefficient of the firm’s ESG_Score on future cash flows is 0.0007, also attaining a 1% significance level. These findings evidently indicate that the firm’s ESG performance exerts a positive influence on both its long-term value and future cash flows.

In the instrumental variable test, the p-value of the underidentification test is less than 0.01, strongly ruling out the possibility of weak identification of the instrumental variable. The F-statistic of the weak instrumental variable test is much larger than the standard value, fully demonstrating the high reliability of the selected instrumental variable. It can effectively alleviate the endogeneity problem and greatly enhance the robustness and credibility of the research conclusions, providing a solid foundation for a deeper understanding of the relationship between ESG and corporate value.

### Further study

#### Is the HHI index above the industry average.

The Herfindahl-Hirschman Index (HHI Index), a key measure of market concentration, is critical for analyzing the relationship between ESG ratings and long-term corporate value. It reveals the competitive structure of a market, offering insights into how firms can leverage ESG for an edge in diverse settings.

In highly concentrated industries, leading firms might emphasize ESG to fortify their standing. Conversely, in competitive markets, firms could use ESG as a differentiation strategy. Table 7 results show that for firms in more competitive markets (HHI index below industry average), the coefficient is 0.0073, significant at 1%. The subgroup test’s empirical p-value for coefficient difference passes significance, suggesting firms in such markets may be more proactive with ESG to stand out, making its positive impact on long-term value more pronounced.

**Table 7:** Grouping regression.

	HHI_high		KeyPollMonUnit		IsPassISO9001	
	0	1	0	1	0	1
ESG_Score	0.0073***	0.0029**	0.0054***	0.0073***	0.0065***	0.0053***
	(0.0009)	(0.0012)	(0.0008)	(0.0014)	(0.0008)	(0.0011)
Board	0.0225***	−0.0063	0.0159***	−0.0046	0.0137**	0.0055
	(0.0069)	(0.0087)	(0.0057)	(0.0130)	(0.0064)	(0.0104)
IndeDir	−0.0760	0.0095	0.0405	−0.5761	−0.0231	−0.3369
	(0.1855)	(0.2694)	(0.1589)	(0.3604)	(0.1777)	(0.2766)
Manahold	−0.0005	−0.0004	−0.0005	0.0002	−0.0004	−0.0006
	(0.0005)	(0.0007)	(0.0004)	(0.0010)	(0.0005)	(0.0007)
Lev	1.1024***	1.1878***	1.0997***	1.2461***	1.1546***	1.0513***
	(0.0608)	(0.0853)	(0.0540)	(0.1131)	(0.0583)	(0.0914)
Size	0.1502***	0.1550***	0.1762***	0.0717***	0.1633***	0.1118***
	(0.0106)	(0.0156)	(0.0098)	(0.0195)	(0.0102)	(0.0173)
Top1	0.0038***	0.0026***	0.0030***	0.0054***	0.0031***	0.0048***
	(0.0006)	(0.0009)	(0.0006)	(0.0012)	(0.0006)	(0.0009)
Growth	0.0164*	0.0258**	0.0147*	0.0308	0.0167**	0.0084
	(0.0085)	(0.0121)	(0.0075)	(0.0226)	(0.0079)	(0.0151)
Lnlistage	−0.0667***	−0.0595***	−0.0842***	0.0443**	−0.0597***	−0.0684***
	(0.0109)	(0.0146)	(0.0096)	(0.0208)	(0.0103)	(0.0158)
CL	0.0761	0.1952***	0.0960**	0.1955*	0.0835*	0.2050**
	(0.0547)	(0.0699)	(0.0480)	(0.1000)	(0.0495)	(0.0871)
PPE	0.1542**	0.2805***	0.2607***	0.0320	0.1852***	0.3150***
	(0.0740)	(0.0949)	(0.0643)	(0.1307)	(0.0678)	(0.1121)
_cons	−2.2584***	−2.1305***	−2.8326***	−0.0661	−2.4996***	−1.2054***
	(0.2387)	(0.3343)	(0.2164)	(0.4385)	(0.2218)	(0.3900)
N	23013	9813	26496	6329	24093	8732
r2_a	0.5063	0.5470	0.5372	0.2683	0.5433	0.4312

Note: *, **, and *** indicate significant at the 10%, 5%, and 1% levels, respectively, and the values in parentheses are cluster robust standard errors.

In contrast, in concentrated industries (HHI index above average), dominant firms, perhaps due to their market power, might be less pressured to boost ESG, potentially weakening the ESG-long-term value link.

#### Key pollution monitoring units.

Whether or not they are labeled as a key pollution monitoring unit by the environmental protection department is used as a grouping criterion. This paper focuses on the ESG performance of such firms. As they usually face stricter environmental regulations and public scrutiny due to their production’s potential environmental impact, it’s hypothesized that their environmental protection-related ESG performance significantly impacts long - term value. The regression results reveal a significant positive relationship, with a coefficient of 0.0073, and the subgroup test’s empirical p - value for coefficient difference passes significance. This indicates that environmental governance investment aids firms in meeting regs and boosts their capital market standing [50].

Heavily polluting firms have higher transformation costs for ESG improvement, but it pays off in the long run. Their continued effective ESG practices can dispel market skepticism, rebuild public trust, and enhance brand image, attracting more stakeholders. Governments often support them for local economic importance. Also, tighter environmental regulations make good ESG performance beneficial for avoiding fines and compliance risks. It further improves operational efficiency, product quality, and competitiveness. In the capital market, it cuts capital costs, optimizes capital structure, and lifts corporate valuation. In sum, by enhancing ESG performance, these firms can meet environmental demands and bolster long - term value for sustainable development.

#### Whether ISO9001 is certified by the system.

The ISO 9001 standard is an internationally recognized standard for quality management systems, and certified companies are considered to have achieved a certain level of quality management and process control. This paper further explores how ISO 9001 system certification affects the relationship between firms’ ESG performance and long-term value. In this paper, firms with ISO 9001 certification information from the CSMAR database are selected as the study sample to compare with non-certified firms. According to the results in Table 7, firms that are not ISO 9001 certified are more significant in the relationship between ESG and long term value of firms with a coefficient of 0.0065 which is significant at 1% level, and the empirical p-value of the coefficient of difference test between the groups passes the test of significance in the group test. The possible reason for this is that for firms that are not ISO 9001 compliant, their quality management systems may not be sufficiently developed, and therefore, even small improvements in ESG may lead to significant marginal improvements in risk management. Resultantly, such improvements can significantly reduce a firm’s operational risk and improve its stability and predictability, thereby positively affecting its long-term value. Non-certified firms may experience higher marginal gains in improving their ESG performance, as the market is likely to place a higher value on these firms’ improvements. This may be due to the fact that investors and consumers have higher expectations for future improvements of these firms. The test results in Table 7 emphasize that the absence of ISO 9001 certification may have weakened the market’s trust in firms. In this case, by improving their ESG performance, firms can demonstrate their commitment to social responsibility and environmental stewardship, and this marginal increase in trust may have a significant positive impact on the long-term value of the firm. In addition, the marginal effect of ESG improvement may be greater for non-certified firms, which provides these firms with opportunities for improvement and upgrading.

### Mechanism testing

In exploring the relationship between firms’ ESG performance and long-term value (ROIC - WACC), we note that ESG practices impact firms’ risks and returns. While prior studies showed ESG’s defensive role in corporate risk, empirical validation of its link to idiosyncratic/systemic risks is lacking. Thus, this study follows the mediating effect testing process [51]. It first examines the ESG - potential mediating variables connection and then theoretically elaborates on the mediating variables’ impact on dependent variables.

Based on prior discussion, we explore ESG’s influence on long-term value and future cash flows, using the Lerner index as a mediator. Corporate ESG performance boosts future cash flows and long-term value by enhancing pricing power. Table 8 shows ESG performance’s regression coefficient on the Lerner Index is 0.0003 and significant. Superior ESG practices may augment brand value and loyalty, granting better market position and pricing freedom, thus increasing the Lerner Index.

**Table 8:** Mechanism testing.

	(1)	(2)	(3)
	Long-Value	FCF	Lerner Index
ESG_Score	0.0059***	0.0003***	0.0003**
	(0.0007)	(0.0001)	(0.0001)
Board	0.0121**	−0.0001	0.0004
	(0.0057)	(0.0004)	(0.0011)
IndeDir	−0.0781	−0.0087	−0.0226
	(0.1573)	(0.0126)	(0.0290)
Manahold	−0.0005	0.0001**	0.0003***
	(0.0004)	(0.0000)	(0.0001)
Lev	1.1300***	−0.0604***	−0.2483***
	(0.0517)	(0.0039)	(0.0102)
Size	0.1523***	0.0072***	0.0244***
	(0.0093)	(0.0007)	(0.0018)
Top1	0.0035***	0.0003***	0.0001
	(0.0006)	(0.0000)	(0.0001)
Growth	0.0150**	−0.0029***	0.0076***
	(0.0072)	(0.0007)	(0.0016)
Lnlistage	−0.0616***	0.0007	−0.0260***
	(0.0091)	(0.0008)	(0.0019)
CL	0.1109**	0.0167***	−0.0810***
	(0.0452)	(0.0035)	(0.0112)
PPE	0.2113***	0.0942***	−0.0506***
	(0.0616)	(0.0047)	(0.0131)
_cons	−2.2184***	−0.1315***	−0.1854***
	(0.2041)	(0.0159)	(0.0448)
N	32826	32826	32826
r2_a	0.5145	0.1130	0.2597

Note: *, **, and *** indicate significant at the 10%, 5%, and 1% levels, respectively, and the values in parentheses are cluster robust standard errors.

There’s a robust link between the Lerner Index and firm’s future cash flows and long-term value. A higher index signals strong pricing and market power, allowing price-setting above marginal cost for greater profitability and cash flows. Such firms stay profitable during fluctuations, enhancing cash flow predictability and long-term value. Also, a high Lerner index indicates entry barriers like tech or branding, ensuring market share and profit sustainability [52]. Ample cash flows offer reinvestment chances, fueling growth.

Overall, the Lerner Index - long-term value (ROIC - WACC) relationship reveals market pricing power’s impact on economic profitability. A high Lerner Index means better profit margins and a positive (ROIC - WACC), showing value creation power. Conversely, a low index signals price constraints and potential capital cost coverage issues. There’s a positive correlation, highlighting pricing power’s role in value creation. This helps firms and investors understand ESG’s financial impact, guiding sustainable growth via optimized ESG strategies.

## Conclusions and recommendations

This paper examines the impact of firms’ ESG performance on long-term value and future cash flows by using a sample of Chinese A-share listed firms from 2007 to 2022. It is found that good ESG performance significantly enhances firms’ future cash flows and long-term value, and the results remain robust after substituting variables and controlling for endogeneity. Specifically, ESG performance enhances capital access by reducing firms’ weighted average cost of capital (WACC) and increasing return on capital (ROIC). In addition, positive ESG inputs help mitigate agency problems, reduce short-term behaviors, promote long-term investment efficiency, and enhance future cash flows, while lowering operating costs and strengthening firms’ risk resistance. The results of the mechanism test indicate that corporate ESG performance improves future cash flows and long-term corporate value by increasing market pricing power, which in turn improves the future cash flows and long-term corporate value of the firm. The results of subgroup regressions show that the positive impact of ESG performance on long-term corporate value is stronger in industries with more competitive markets, in key pollution-monitored firms, and in firms that are not ISO 9001 certified.

### Policy promotion and strategic adaptation

The Chinese government actively advocates the concept of sustainable development. Policy orientations such as the “dual-carbon” goal and common prosperity prompt enterprises to incorporate ESG into their long-term strategic planning. From a macro perspective, a series of policies that encourage green development, social responsibility fulfillment, and corporate governance improvement have been successively introduced, providing clear directions and policy support for enterprises to practice ESG. For example, support policies for the environmental protection industry encourage enterprises to increase their investment in areas such as energy conservation, emission reduction, and clean energy development. This not only helps enterprises meet policy requirements but also enables them to enhance their long-term competitiveness through technological innovation and industrial upgrading, thereby increasing the long-term value of the enterprise. If enterprises actively respond to policy calls and layout ESG strategies early, such as adjusting the industrial structure towards green and low-carbon transformation, although they may face an increase in short-term costs, in the long run, they are expected to gain an advantageous position in the emerging green market, obtain policy dividends and first-mover advantages in the market, and achieve steady growth in long-term value.

We found that enterprises actively implementing ESG strategies exhibited a more positive trend in long-term value indicators (ROIC - WACC). Taking some enterprises actively involved in green technology research and development investment as an example, although a large amount of capital was required for equipment procurement and technical talent recruitment in the initial stage of research and development, with the gradual maturity and application of technology, the enterprise’s energy utilization efficiency was significantly improved, and the energy consumption cost per unit of product was greatly reduced. Meanwhile, with the increase in market demand for green products, the enterprise’s product market share gradually expanded, and sales revenue continued to grow, thereby driving the improvement of ROIC. Due to its positive performance in sustainable development, the enterprise’s image in the capital market was improved, and investors’ risk assessment of it was reduced, enabling the enterprise to obtain more favorable interest rate conditions in the financing process and thus reducing the WACC. This process fully reflects that under the Chinese policy environment, the improvement of enterprise ESG performance has a positive impact on long-term value by affecting ROIC and WACC.

### Market perception and investor-driven

With the continuous development and maturity of the Chinese capital market, investors’ attention to enterprise ESG performance has gradually increased. More and more institutional investors have begun to incorporate ESG factors into the investment decision-making process and show more preference for enterprises with good ESG performance. This market trend prompts enterprises to attach importance to ESG work in order to attract long-term and stable investment. In the research sample of this article, we observed that with the gradual popularization of the ESG concept in the capital market, there is a significant positive correlation between enterprise ESG performance and market valuation. Especially for those enterprises with outstanding performance in environmental information disclosure, investors are more optimistic about their future development prospects and are willing to pay a higher price for their stocks. This phenomenon indicates that under the Chinese market environment, good ESG performance of enterprises can effectively attract investors’ attention and capital inflows, providing strong financial support for the long-term value growth of enterprises. At the same time, through active ESG practices such as participating in social welfare activities and improving employee benefits, enterprises enhance their social image and brand value, further improving their competitiveness and long-term value in the market.

In summary, under the actual situation in China, enterprise ESG performance has a profound impact on its long-term value through the comprehensive effects of policy guidance, market mechanisms, industry competition, consumer demand, and social environment. Enterprises should fully recognize this trend and actively integrate the ESG concept into all aspects of enterprise operation and management to achieve long-term sustainable development and value maximization. The limitation of this paper’s research is that in the presence of ESG rating divergence, there is currently no clear answer in academia about the optimal ESG rating results, and this paper mainly adopts the ESG rating results provided by the CNRDS database as a proxy variable for firms’ ESG performance, which may be subject to measurement error. Future research can delve into the impact of corporate ESG performance on corporate value creation in the presence of ESG rating divergence.
